# Undetected tuberculosis at enrollment and after hospitalization in medical and oncology wards in Botswana

**DOI:** 10.1371/journal.pone.0219678

**Published:** 2019-07-11

**Authors:** Yeonsoo Baik, Othusitse Fane, Qiao Wang, Chawangwa Modongo, Cynthia Caiphus, Surbhi Grover, Nicola M. Zetola, Sanghyuk S. Shin

**Affiliations:** 1 Department of Epidemiology, UCLA Fielding School of Public Health, Los Angeles, CA, United States of America; 2 Botswana-Upenn Partnership, Gaborone, Botswana; 3 Sue & Bill Gross School of Nursing, University of California, Irvine, Irvine, CA, United States of America; 4 Department of Infectious Disease, University of Pennsylvania School of Medicine, Philadelphia, PA, United States of America; 5 Princess Marina Hospital, Botswana Ministry of Health, Gaborone, Botswana; 6 Department of Radiation Oncology, University of Pennsylvania School of Medicine, Philadelphia, PA, United States of America; 7 Department of Critical Care Medicine, University of Maryland Medical Center, Baltimore, MD, United States of America; The University of Georgia, UNITED STATES

## Abstract

Cancer patients are at higher risk of tuberculosis (TB) infection, especially in hospital settings with high TB/HIV burden. The study was implemented among adult patients admitted to the largest tertiary-level referral hospital in Botswana. We estimated the TB prevalence at admission and the rate of newly diagnosed TB after hospitalization in the medical and oncology wards, separately. Presumptive TB cases were identified at admission through symptom screening and underwent the diagnostic evaluation through GeneXpert. Patients with no evidence of TB were followed-up until TB diagnosis or the end of the study. In the medical and oncology wards, four of 867 admitted patients and two of 240 had laboratory-confirmed TB at admission (prevalence = 461.4 and 833.3 per 100,000, respectively.) The post-admission TB rate from the medical wards was 28.3 cases per 1,000 person-year during 424.5 follow-up years (post-admission TB rate among HIV-positive versus. -negative = 54.1 and 9.8 per 1,000 person-year, respectively [Rate Ratio = 5.5]). No post-admission TB case was detected from the oncology ward. High rates of undetected TB at admission at both medical and oncology wards, and high rate of newly diagnosed TB after admission at medical wards suggest that TB screening and diagnostic evaluation should target all patients admitted to a hospital in high-burden settings.

## Introduction

Nosocomial transmission of tuberculosis (TB) has remained a persistent problem in low resource settings.[1, 2] Overcrowding and limited implementation of infection control practices make healthcare facilities highly vulnerable to TB transmission.[3] TB transmission in healthcare facilities not only puts healthcare workers at higher risk of TB but can also lead to increased TB transmission in the community after infected patients are discharged from the hospital.[2] Consequently, health facilities may serve as important drivers of the TB epidemic in the general population.[1, 4]

Nosocomial TB transmission is a greater concern in areas where a high proportion of people who seek care are susceptible to TB, such as HIV/AIDS patients and patients receiving cancer treatment.[1, 5, 6] Immunocompromised status among HIV positive patients increases the risk of disease progression to active TB once they are infected. Patients with cancers are also immunosuppressed as a combination of their primary diseases and cancer-related treatments.[5, 7, 8] In addition to the possible biological relationship, shared risk factors with TB such as smoking, alcohol consumption, and chronic obstructive pulmonary disease may increase TB risk among cancer patients. However, there is still a dearth of information on the burden of TB disease among cancer patients, especially in high HIV and TB burden countries.[7] Accordingly, TB cases may have been missed in routine practice because of lower suspicion of TB in those target populations. No other previous studies have conducted active case finding in oncology ward, nevertheless.[5]

In this study, we explored the burden of TB disease among hospitalized patients at the largest main tertiary-level referral hospital, Princess Marina Hospital, in Gaborone, Botswana. We first aimed to compare the TB prevalence among patients admitted to the medical and oncology wards at the day of their admission. Second, we estimated the rate of newly diagnosed TB cases presenting after hospitalization, or post-admission TB rate, in medical and oncology wards. In this study, we did not use the terms ‘incident’ or ‘co-prevalent’, because we could not confirm with molecular epidemiology. We instead indicated the TB cases identified after hospitalization as ‘post-admission’ or ‘newly diagnosed after admission’. We also compared the relative risk of post-admission TB rate among HIV-positive and HIV-negative patients.

## Materials and methods

### Study setting

Botswana is a country of hyper-endemic for TB and HIV, with the TB incidence rate of 326 per 100,000 and about 70% of TB patients were co-infected with HIV/AIDS.(9, 10) According to the Botswana National TB Program (BNTP) guidelines, all patients aged 15 years or older are screened for TB symptoms at healthcare facilities on their admission. Patients reporting cough of any duration, night sweats, chest pain, or weight loss during the prior month are considered presumptive TB cases. Presumptive TB patients provide one sputum specimen, self-produced or induced, for TB diagnostic evaluation by GeneXpert MTB/RIF (Xpert), as recommended by the World Health Organization (WHO). The Xpert has been the primary diagnosis tool since 2016 in Botswana. Presumptive TB patients whose Xpert does not detect *Mycobacterium tuberculosis* undergo Xpert testing a second time together with clinical assessment and chest X-ray. All diagnosed TB patients start on TB therapy according to the BNTP guidelines.

### Study population

Between August 2016 and December 2017, we recruited patients who were admitted to the general medical and oncology wards at the Princess Marina Hospital. All patients in the oncology ward had a biopsy-proven cancer diagnosis. All patients in the hospital aged 15 years or older were screened for TB symptoms at admission. Patients who were non-presumptive TB cases and who were ruled out for TB after confirmatory testing were enrolled for a follow-up study. Enrolled patients were screened daily for TB symptoms during their stay at the hospital and the diagnostic evaluations were performed as indicated by the attending physician. They were technically followed up to 21 months, from their admission to April 2018, however, the final follow-up was 18 months, counted from August 2016 until January 2018. We assumed a three-month of lag time between TB occurrence and the time it would be captured at the BNTP database. We accounted the lag time for person-time calculation by subtracting three months from the actual end period of the follow-up.

### Laboratory procedures

One sputum specimen per patient was voluntarily collected from presumptive TB patients and evaluated with Xpert or smear microscopy at the Princess Marina Hospital Clinical Laboratory. Only when the Xpert testing was not possible due to stock out or maintenance issues, the smear microscopy replaced the Xpert.

### Demographic and clinical measures

At baseline, patients’ characteristics were collected through face-to-face interviews and abstraction of electronic- and paper-based medical records. Information regarding demographic characteristics, HIV status, treatment by antiretroviral therapy, malignancies, immune-suppressive therapies, and dates of hospitalization were collected. Rapid HIV testing was performed for all persons presenting to the hospital as a part of routine care, following the Botswana HIV testing algorithms as recommended by the WHO. [9, 10]

### Ascertainment of post-admission TB during the follow-up

The patients, who had no evidence of TB at the time of their admission, were followed until diagnosed as TB or by the end of the study protocol (April 30th, 2018), whichever came first. Of those patients, we excluded the patients who did not agree with participation in the study, those whose assigned ward was missing, those whose TB diagnosis date, treatment date, or discharge date was before or on the time of hospital admission, and those whose discharged data had TB records but no diagnosis date. The patients who have developed TB during the hospitalization or after discharge were considered the post-admission TB cases. To identify TB cases occurring after their discharge, we matched our database with the BNTP database by patients’ national identification number and their name at the end of the follow-up period. To capture any missing cases and extra-pulmonary TB cases, we also reviewed the medical record when the patients were discharged.

### Statistical analyses

Patients’ characteristics were described by percentages for categorical variables and means and standard deviations for continuous variables. The prevalence at admission was calculated by the number of TB cases on admission through the screening process divided by the total number of admitted patients who went through the symptom-based screening during the study period. The post-admission TB rate was calculated as the number of new TB cases identified after admission and during the study period, divided by the sum of person-times of all patients who were at risk. The post-admission TB rate was estimated within HIV positive and negative populations separately, and compared by calculating the rate ratio. We calculated 95% confidence intervals (CI) for TB prevalence at admission, the post-admission TB rates, and the rate ratio. TB cases’ person-times were interval days from hospital admission to TB diagnosis. Non-TB cases’ person-times were interval days between hospital admission and three months (90 days) prior to the end of the study protocol, January 30^th^, 2018. The entire patient population’s and the TB patients’ median hospitalization period was also estimated, when both admission and discharge dates were reported. All statistical analyses were performed using R version 3.0.4. (The R Project for Statistical Computing; www.r-project.org)

### Ethical considerations

The Botswana Ministry of Health Human Research Development Committee, the Princess Marina Hospital Ethical Committee, the University of Pennsylvania IRB, and the University of California Los Angeles IRB approved the study. The written informed consent was obtained from the all participants.

## Results

**Fig 1:**
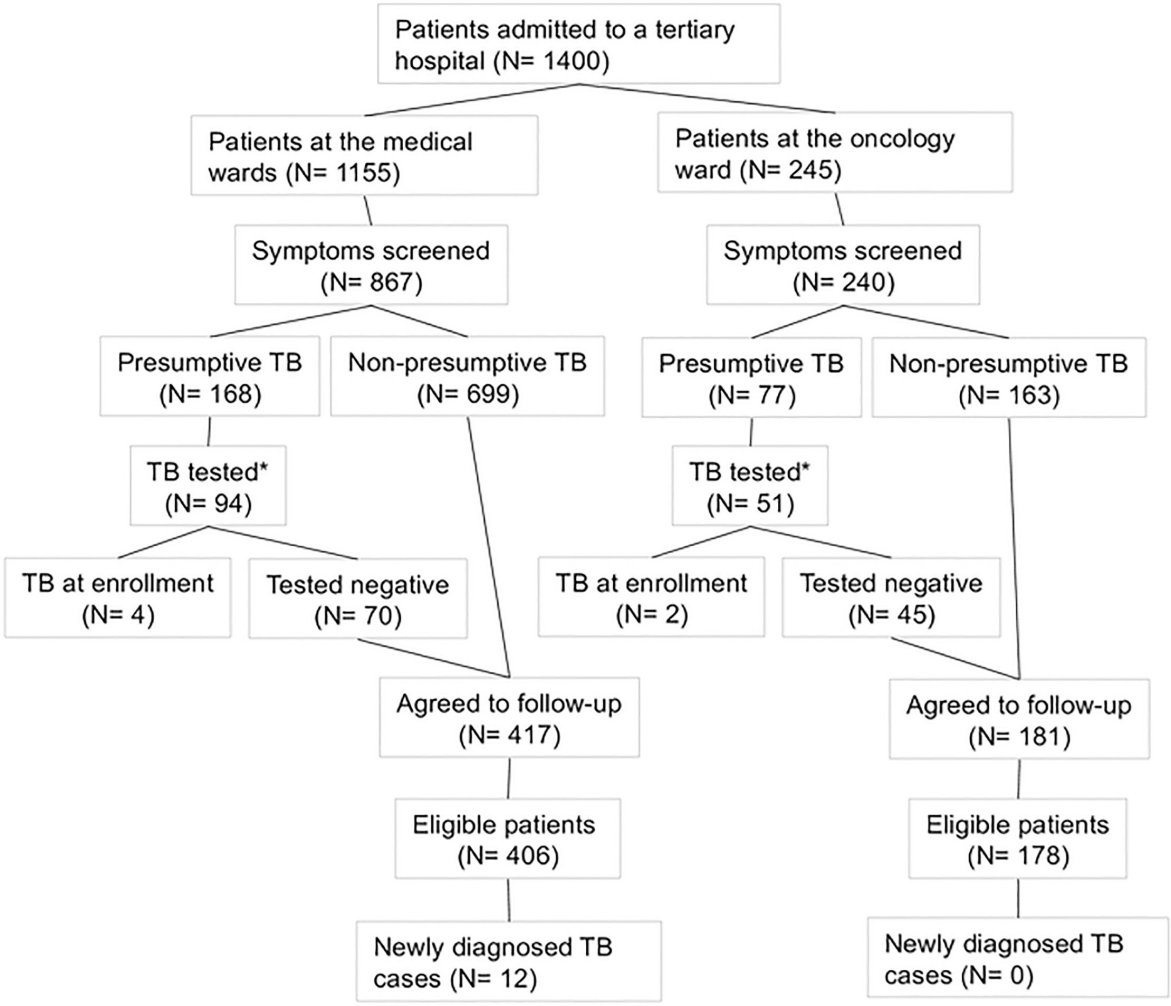
Study flow chart. The number of not available (NA) test result = 20 in the medical and 4 in the oncology wards. TB; tuberculosis.

During the recruitment period, there were 1,400 patients admitted to the hospital; 1,155 (82.5%) to the medical wards versus 245 (17.5%) to the oncology ward. In the medical and the oncology wards respectively, 867 (75%) and 240 (98%) of admitted patients went through the symptom screening process. The patients screened for TB symptoms at admission were older from the oncology ward, and a higher proportion were female and HIV infected. (Table 1) Among the patients who were screened at the medical and oncology wards, 168 (19%) and 77 (32%) reported presumptive TB symptoms; 94 (56%) and 51 (66%) were microbiologically tested for TB; and 4 (4%) and 2 (4%) were tested positive for TB, respectively. This corresponds to a yield of lab-confirmed TB at admission of 0.5% (4/867) or 461.4 per 100,000 (95%CI = 146.4–1109) and 0.8% (2/240) or 833.3 per 100,000 (95%CI = 139.6–2726) in the medical and oncology wards, respectively. (Fig 1) Among the six prevalent TB cases, two patients from the medical wards were HIV positive.

**Table 1:** Characteristics of study population who were screened for presumptive tuberculosis symptoms between August 2016 and June 2017 (N = 1107).

	Medical wards	Oncology ward
	(N = 867, 78%)	(N = 240, 22%)
	N (%)	N (%)
*Age (years)* *	41.9 ± 18.5	53.5 ± 16.4
*Gender*		
Female	495 (57%)	151 (63%)
Male	372 (43%)	89 (37%)
*HIV status*		
Positive	327 (38%)	113 (47%)
Negative	431 (50%)	107 (45%)
Unknown	109 (13%)	20 (8%)
*Presumptive TB*	168 (19%)	77 (32%)
*Laboratory-confirmed TB*^†^	4 (4%)	2 (4%)
*TB Prevalence (per 100*,*000)*	461.4	833.3

* Mean ± Standard Deviation

^†^ % was estimated out of the number of microbiologically tested presumptive cases, which was 94 and 51in the medical and oncology wards, respectively

Overall, 406 (97%) and 178 (98%) eligible patients from the medical and the oncology wards, respectively, were included as the final follow-up population. The total follow-up time was 617.1 person-years, counted from August 2016 until January 2018; 424.5 and 192.6 person-years in the medical and the oncology wards, respectively. The patients from the oncology ward were more likely to be older, HIV infected, and on ART than medical wards. (Table 2) Over 60% of the participants from both wards were female patients.

**Table 2:** Characteristics of follow-up cohort who were non-presumptive tuberculosis on enrollment (N = 584).

	Medical wards	Oncology ward
	(N = 406, 70%)	(N = 178, 30%)
	N (%)	N (%)
Age (years)*	40.4 ±16.1	49.4 ±15.5
*Gender*		
Male	160 (40)	67 (38)
Female	243 (60)	109 (62)
*HIV*		
Positive	158 (39)	85 (48)
+ Antiretroviral therapy	135 (85)	80 (94)
- Antiretroviral therapy	21 (13)	4 (5)
Negative	200 (50)	70 (40)
Unknown	45 (11)	22 (12)
*Chemotherapy*	1 (0)	34 (19)
*Radiation therapy*	2 (0)	20 (11)
*Newly diagnosed TB*	12 (3)	0
HIV positive	9 (75)	0
*Type of cancer by location*		
Breast	-	13 (7)
Digestive/Gastrointestinal	-	23 (13)
Gynecologic	-	32 (18)
Head and Neck	-	15 (8)
Hematologic/Blood	-	8 (4)
Respiratory/Thoracic (lung)	-	4 (2)
Skin	-	7 (4)
Others^†^	-	10 (13)
Unknown		66 (37)
*Post-admission TB rate (cases/1*,*000 person-year)*	28.3	0
*Overall post-admission TB rate (cases/1*,*000 person-year)*	19.4	

* Mean ±Standard Deviation

^†^ Other cancer types by location include bone (1), eye (1), germ cell (2), genitourinary (4), and musculoskeletal (2)

There were 12 post-admission TB cases identified from the medical wards, nine (75%) of whom were HIV positive, whereas no post-admission TB were identified from the oncology ward. (Table 3) All 12 TB cases were initially considered non-presumptive TB cases. Among those, nine cases were diagnosed during hospitalization and identified by the hospital records, eight (89%) were HIV positive. Three cases were diagnosed as TB after discharge, identified through the BNTP database. The overall post-admission TB rate was 19.4 cases per 1,000 person-year (95% CI = 10.5–33.1 cases per 1,000 person-year). The post-admission TB rate from the medical wards was 28.3 cases per 1,000 person-year (95% CI = 15.3–48.1 cases per 1,000 person-year). The post-admission TB rate during the hospitalization included nine TB cases identified during 132.7 person-years of follow-up, was 67.8 cases per 1,000 person-year (95% CI = 33.1–124.5 cases per 1,000 person-year).

**Table 3:** Newly diagnosed tuberculosis patients (N = 12).

	Age (years)	Gender	Previous TB treatment	HIV status	Antiretroviral therapy history	Interval days*
Patient A	31	Male	No	Positive	Yes	6
Patient B	34	Male	No	Positive	Yes	18
Patient C	32	Male	No	Negative	NA	36
Patient D	34	Male	No	Positive	Yes	14
Patient E	41	Male	Yes	Positive	Yes	1
Patient F	33	Male	No	Positive	Yes	4
Patient G	35	Female	No	Positive	Yes	10
Patient H	57	Male	Yes	Unknown	NA	11
Patient I	47	Male	No	Positive	Yes	1
Patient J	34	Male	No	Positive	Yes	10
Patient K	40	Female	No	Negative	NA	35
Patient L	49	Male	No	Positive	Yes	5

* Interval days between admission date and time of diagnosis

NA; Not Applicable

The median time of hospitalization for the follow-up population was 9 days (interquartile range [IQR] = 5–15) in the medical wards among 324 patients and 22 days (IQR = 11–32) among 102 patients in the oncology ward. The median time between TB diagnosis and the hospital admission was 10 days (IQR = 4–14) in the medical wards; 6 days (IQR = 4–10) among nine HIV-positive and 35.5 days among two HIV-negative patients (35 and 36 days).

**Fig 2:**
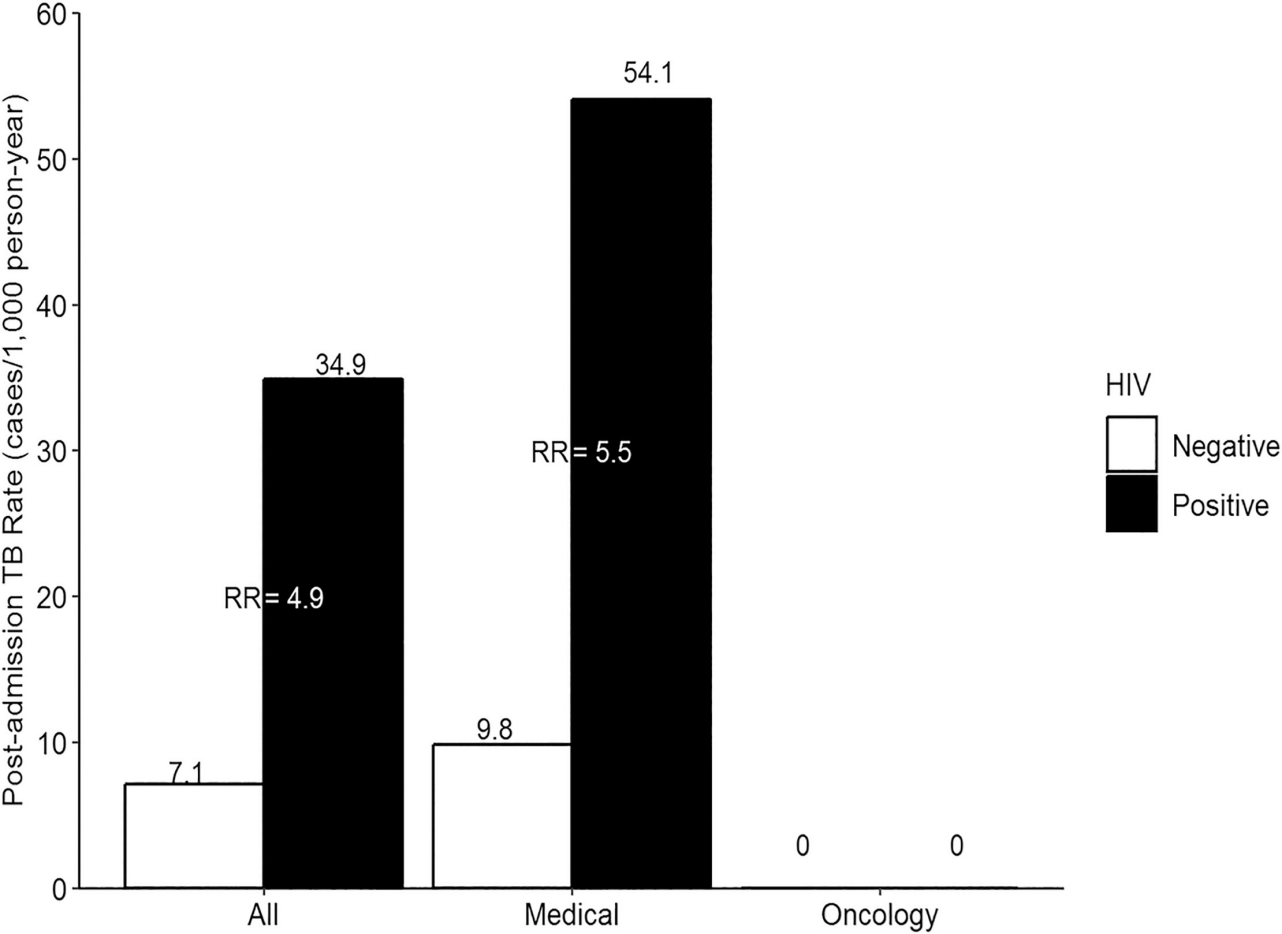
Rate ratio of tuberculosis from HIV positive versus HIV negative. RR; Rate Ratio, TB; tuberculosis. The post-admission TB rates were estimated with 11 newly diagnosed TB cases (9 were HIV positive and 2 were HIV negative) after excluding one case whose HIV status was unknown.

The post-admission TB rates among HIV positive and negative patients were 34.9 cases per 1,000 person-year (95% CI = 17.0–64.0) and 7.1 (95% CI = 1.2–23.6), respectively. The post-admission TB rate ratio among HIV positive to HIV negative patients was 4.89 (95% CI = 1.2–33.2) In the medical wards only, the post-admission TB rates among HIV positive and negative patients were 54.1 cases per 1,000 person-year (95% CI = 26.4–99.3) and 9.8 (95% CI = 1.7–32.5), respectively. Its rate ratio was 5.5 (95% CI = 1.3–37.4). (Fig 2)

## Discussion

We found the high prevalence of TB on admission among patients who were admitted to the hospital, particularly from the oncology ward. A thorough screening and microbiologic diagnosis were critical for identifying TB cases that may have gone undetected if not by our screening program (no active pulmonary TB cases were identified in the oncology ward during the year prior to the implementation of this study). Our finding of high prevalence of TB in the oncology ward at admission aligns with previous reports of high co-existence rate of TB and lung cancer.[8, 11] Future studies should investigate whether a causal relationship exists between TB and cancer.[11–16]

To our knowledge, our study was the first study of active TB case finding in the oncology ward. Currently the systematic screening for active TB is recommended among household contacts especially young children, people living with HIV at their visit to healthcare, and workers exposed to silica (or miners).[17] Diagnostic yields (number of TB cases diagnosed / number screened) in these populations were estimated about 2%.[18] In contrast, the number that needed to be screened to identify one TB case was known to be larger in hospital settings. Though it varied across different populations, the yield was 0.7% in the previous active case finding study in outpatient hospitals in Ghana.[18, 19] Our case finding yielded <1% in both wards. Our yield may have been affected by low coverage of diagnostic testing as only 56% in the medical wards and 66% in the oncology wards underwent the TB testing after showing presumptive TB symptoms. While we sought to perform microbiological testing of all patients with TB symptoms, many patients were too ill to provide sputum samples when approached by our research staff. This finding highlights the difficulty in achieving high rates of TB testing in real-life inpatient settings. The lower yield may also be resulted from highly immunosuppressed patients who frequently experienced producing less amount of sputum or paucibacillary TB.[20] The laboratory confirmation may have been challenge in these cases. Therefore, additional research is needed to determine the most effective TB screening algorithm in this population.[18]

We also found high post-admission TB rate after discharge from the hospital (19.4 cases per 1,000 person-year), especially from the medical wards (28.3 cases per 1,000 person-year). It represented a 6–9 fold higher compared to 3.26 per 1,000 persons of the annual TB incidence in Botswana in 2016.[21] This high TB rate after hospitalization was aligned with the previous study findings in a similar hospital setting in Botswana, which identified about 37 cases per 1,000 person-year. [2] We were not able to detect any newly diagnosed TB from the oncology ward. The lack of detection was highly likely from patients’ high mortality during the follow-up and high rates of existing extra-pulmonary TB. Our sample size may have been too small and follow-up duration too short for detecting newly diagnosed TB.

Previous studies, though mostly were conducted in low TB-burden countries, presented the increased relative risk of TB infection among cancer patients compared to the general population. The risk varied over a wide range, depending on a type of cancer, population, and time since diagnosis of cancer.[5, 7, 22] It was reported high at the time of diagnosis of hematological, head and neck, or solid caners (e.g. lung cancer) in adults in Denmark, Taiwan, Korea, and South Africa, whereas in the US-born population the risk did not increase with solid tumors.[5, 7, 22]

We performed TB screening and laboratory-based diagnostic evaluation targeting all patients admitted to the hospital who had presumptive symptoms; however, it is possible that patients who denied having symptoms and did not go through any diagnostic evaluations actually had TB at baseline but were diagnosed later. Indeed, our data showed a short median interval days from hospital admission at the medical wards to diagnosis time, 10 out of 12 cases were diagnosed as TB less than one month after hospital admission. Given that the incubation period of active TB cases is believed to be around 3–9 months, [23] these post-admission TB cases may have been missed at admission. As we mentioned, without molecular epidemiologic confirmation, we were not certain whether the 12 post-admission TB cases were ‘incident’ after admission or ‘co-prevalent’ on admission. However, as all 12 cases were non-presumptive during the screening on admission but presented TB shortly after admission, the symptom-based TB screening may not have sufficient sensitivity to adequately detect TB in health facilities. In high risk settings, universal Xpert-based TB screening for all admitted TB patients should be warranted to diagnose unsuspected TB cases and to prevent secondary transmission.

Our study had several limitations. First, we are likely to have missed TB cases who died from TB-related causes. The competing risks may have led to fewer opportunities for reactivation of TB acquired in the hospital. This scenario may have been more common among cancer patients, whose life expectancy is reduced. [5] Considering that HIV-positive patients dying from TB would be classified as dying from HIV according to the International Classification of Diseases (ICD),[24] both competing risks and disease misclassification may significantly affect the finding in our study, which was conducted in the HIV-prevalent setting. In addition to the mortality among TB/HIV co-infected patients, the morbidity may affect the finding of few newly diagnosed TB cases. The Xpert had lower sensitivity to detect TB among HIV positive patients than the culture diagnosis, though the Xpert is currently the primary diagnosis tool recommended by the WHO. Regardless of repeated application of the Xpert on the second sputum sample, together with chest X-ray and clinical diagnosis, when the first result was negative, TB infection among HIV-positive patients was likely extra-pulmonary, which were not able to be identified based on our study procedures, unless recorded in the discharge form. Despite extra-pulmonary TB case’s less infectiousness, it may lead to underestimation of the burden of TB overall. Furthermore, while we collected type of cancer (63%) and its treatment (11–19%), there were high proportions of missing and we did not collect duration of morbidity. The duration of cancer morbidity is important, as the risk of active TB may be high shortly after a cancer diagnosis and lower after time.[7] Cancer therapies may also affect the risk of reactivation of TB differently over time and depending on the type of cancer.[6] Lastly, we were not able to screen all eligible patients identified during the study period due to several factors such as logistical difficulties of conducting screening among severely ill patients or those quickly discharged and/or transferred. Even the follow-up process after patients’ discharge, by matching the discharged patient list to the BNTP database, may prevent from catching new TB cases. We noticed a few identification information entered incorrectly through the matching process., and, this could have underestimated newly diagnosed TB cases.

Our study findings have several implications. First, strict adherence to TB screening guidelines was critical for timely case detection.[5] Not only benefit to TB patients, does rapid diagnosis of TB also reduce the risk of hospital-associated transmission.[4, 25, 26] This is particularly critical in settings like Botswana where populations are heavily affected by HIV and cancer. If patients were to be detected at enrollment, it would be important to isolate patients and follow the infection control measures to prevent hospital transmission. For instance, in case of our study, the TB patients identified and promptly treated at admission from the oncology ward may have prevented further TB transmission to other vulnerable cancer patients. Future studies applying molecular epidemiology are suggested to confirm whether post-admission TB cases are truly newly diagnosed TB due to nosocomial transmission or co-prevalent TB cases that could have been undetected at admission. Furthermore, to prevent secondary transmission after discharge, diagnostic prediction methods should be developed to assess whether patients can be discharged safely to the community.[27]

## Conclusions

We found high rates of undetected TB at admission from both the medical and oncology wards. Post-admission TB rate after hospital admission was also high in the medical wards. We recommend laboratory-based TB screening and diagnostic evaluation should target all patients admitted to hospitals, and future research apply molecular epidemiology to confirm nosocomial transmission.
